# A Content Review of Life Cycle Assessment of Nanomaterials: Current Practices, Challenges, and Future Prospects

**DOI:** 10.3390/nano11123324

**Published:** 2021-12-07

**Authors:** Nurul Umairah M. Nizam, Marlia M. Hanafiah, Kok Sin Woon

**Affiliations:** 1 Department of Earth Sciences and Environment, Faculty of Science and Technology, Universiti Kebangsaan Malaysia, Bangi 43600, Selangor, Malaysia; p104399@siswa.ukm.edu.my; 2 Centre for Tropical Climate Change System, Institute of Climate Change, Universiti Kebangsaan Malaysia, Bangi 43600, Selangor, Malaysia; 3 School of Energy and Chemical Engineering, Xiamen University Malaysia, Jalan Sunsuria, Bandar Sunsuria, Sepang 43900, Selangor, Malaysia; koksin.woon@xmu.edu.my

**Keywords:** life cycle assessment, environmental indicator, nanoparticles, sustainability, green chemistry

## Abstract

This paper provides a comprehensive review of 71 previous studies on the life cycle assessment (LCA) of nanomaterials (NMs) from 2001 to 2020 (19 years). Although various studies have been carried out to assess the efficiency and potential of wastes for nanotechnology, little attention has been paid to conducting a comprehensive analysis related to the environmental performance and hotspot of NMs, based on LCA methodology. Therefore, this paper highlights and discusses LCA methodology’s basis (goal and scope definition, system boundary, life cycle inventory, life cycle impact assessment, and interpretation) to insights into current practices, limitations, progress, and challenges of LCA application NMs. We found that there is still a lack of comprehensive LCA study on the environmental impacts of NMs until end-of-life stages, thereby potentially supporting misleading conclusions, in most of the previous studies reviewed. For a comprehensive evaluation of LCA of NMs, we recommend that future studies should: (1) report more detailed and transparent LCI data within NMs LCA studies; (2) consider the environmental impacts and potential risks of NMs within their whole life cycle; (3) adopt a transparent and prudent characterization model; and (4) include toxicity, uncertainty, and sensitivity assessments to analyze the exposure pathways of NMs further. Future recommendations towards improvement and harmonization of methodological for future research directions were discussed and provided. This study’s findings redound to future research in the field of LCA NMs specifically, considering that the release of NMs into the environment is yet to be explored due to limited understanding of the mechanisms and pathways involved.

## Highlights

- A total of 71 studies on life cycle assessment of nanomaterials application were reviewed.
- Environmental performance and hotspot of nanomaterials were identified.
- Challenges and prospects for life cycle assessment of nanomaterials were discussed.
- Only five studies considered the exposure pathway of the nanomaterials.
- Of all the studies, 92% neglect the uncertainty analysis within the LCA.

## 1. Introduction

**Figure 1:**
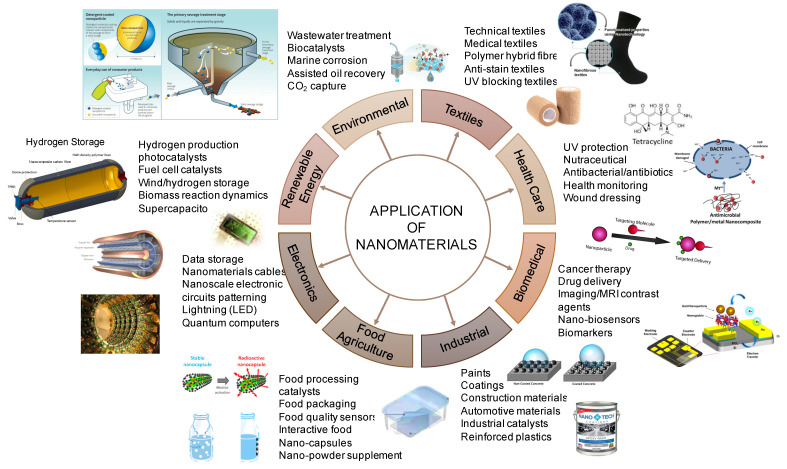
The application of nanomaterials in various sectors.

The use of nanomaterials (NMs) in various applications, including those in biomedical and healthcare, textile industry, environment, agriculture, electronics, energy, and construction and building sectors, have emerged in the past few years, as shown in Figure 1 [1,2,3]. Nanotechnology has attracted a significant discovery towards novel applications incorporating NMs due to its high-performance materials, significant commercial impacts, energy storage and conversion capability, cost and energy savings, and reduced environmental impacts [1,4].

NMs are used in the technology that exists in various forms, such as single, fused, aggregated, tubular, and irregular shapes, and various types, including nanotubes, quantum dots, films, plates, and fullerenes [5]. Due to their unique physical, chemical, mechanical, and efficacy characteristics, there is a growing interest in NMs production. One of the novel properties of NMs is the physical behavior that changed from classical physics to quantum physics with decreasing particle size, in this case between 1 and 100 nm. The size effect of NMs renders high surface energy, a large fraction of surface atoms, and spatial confinements [6]. The unique properties of NMs are their quantum effects, relating to the domination of the matter’s behavior at the nanoscale affecting the optical, electrical, and magnetic behavior of materials [6,7]. Due to these properties, the NMs are increasingly applied in various fields, including environmental remediation, mechanical, and electronic fields (mainly as a catalyst), as shown in Figure 1. Some of the typical applications of NMs that benefit the environment are on-site remediation and wastewater treatment, nanomaterial-based solar cells for improved energy efficiency, and as nanostructured filters or membranes for water purification and air purification [4].

Although many benefits of NMs applications in various sectors have been reported, the widespread use of NMs in development and applications may exhibit potential health and environmental risks which might not yet be fully understood [4,8,9]. The production of NMs usually employs bottom–up processes, such as physical and chemical vapor deposition and activation, carbonization, liquid-phase synthesis, and self-assembly, most of which require massive energy and material inputs that eventually produce pollutants, in terms of effluents and emissions to air, water, and soil [6,10]. Thus far, most research on NMs has focused on their unique functionality in different fields and applications without considering the potential environmental effects throughout their life cycle [11,12,13]. There is also a concern on the environmental sustainability of NMs pathways contributing to environmental problems [11,14,15,16,17].

**Figure 2:**
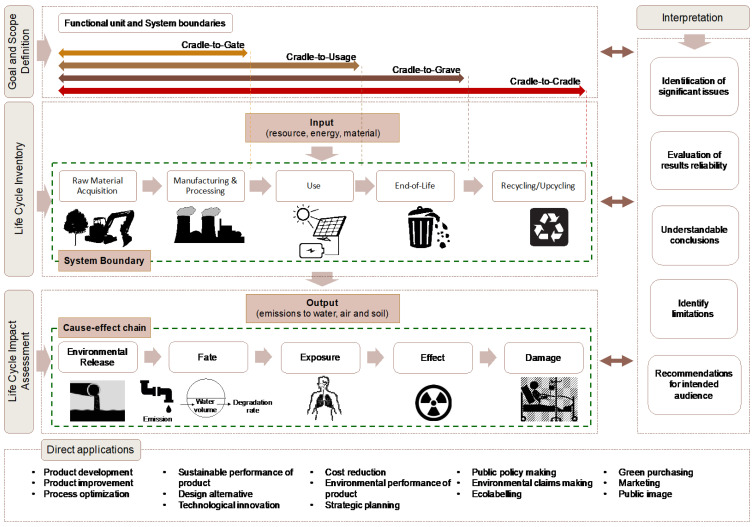
A generic life cycle assessment framework.

Thus, a comprehensive tool, such as life cycle assessment (LCA), can provide better understanding of the potential environmental problems and ensure the environmental sustainability of NMs [12,18]. LCA is a holistic approach to assess environmental impacts of a product throughout its entire life cycle by identifying the materials used and energy and emissions released to the environment [13,19,20,21], which is crucial in evaluating the potential impacts of nanomaterial releases, as shown in Figure 2. LCA is an international standardized methodology, based on the International Organization for Standardization (ISO) 14040 series (ISO 2006; 2006; 2006; 2006d), comprising four phases, as follows: (i) goal and scope, (ii) life cycle inventory, (iii) life cycle impact assessment, and (iv) life cycle interpretation (Figure 2). This methodology was developed as a tool to assess the environmental impact of products, and the processes associated with these products [19,22,23,24,25].

Previous studies have been carried out on LCA of NMs and have found that there are three main challenges that arise when modeling nanomaterials in the LCA framework [11], namely: (1) insufficient use of a proper and adequate functional unit that takes into account all the detailed and additional functionalities of NMs; (2) lack of transparent life cycle inventory (LCI) data in the production of NMs, where materials and energy inputs are often not provided by manufacturers due to the commercial confidentiality; and (3) lack of characterization methods for released NMs, which are a crucial part within the LCIA context.

Due to the rapid technological advancement in nanotechnology, the environmental toxicity pathways of the NMs deserve further investigation from an LCA perspective. However, the methodological approach used, the data collection methods, and the chosen characterization methods in most studies in the field are not consistent, and hence the results are not convincing or might be contradictory to each other. Therefore, the current state-of-the-art LCA application in nanotechnology needs to be explored to gain insights into the current practices of LCA application in nanotechnology and its future outlook. In the present study, content analysis is used to categorize the existing studies on this topic, based on the four phases the LCA comprises. It is important to note that this review topic is essential to highlight the current practices, challenges, and progress to provide recommendations for future studies of LCA applications on NMs.

## 2. Life Cycle Assessment of Nanomaterials

### 2.1. Inclusion of Existing LCA Studies

**Figure 3:**
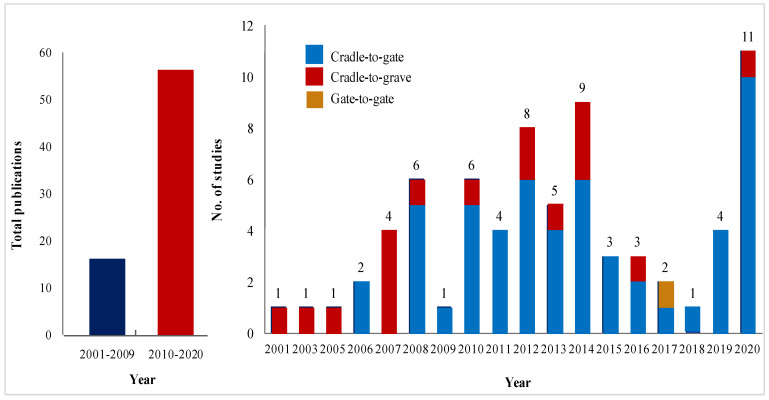
Distribution of papers based on the year and system boundary.

Broad search engines and databases such as Springer, Google Scholar, and Science Direct were used to ensure a complete search of relevant literature. Different branches and names of nanotechnology, such as nanomaterials, nanocomposites, nanobots, and nanoparticles, were included during the search. In addition, keywords, such as life cycle assessment, life cycle analysis, environmental impacts, and environmental evaluation, relevant to nanotechnology, were considered; therefore, the literature search was performed using keywords of life cycle assessment of nanomaterials, life cycle analysis of nanomaterials, environmental impacts of nanomaterials, and environmental evaluation of nanomaterials. Initially, 182 studies were found; however, the numbers were reduced to 126 studies, considering only studies published in scientific indexed journals. To ensure the LCA and nanomaterials relevance, only the literature with a focus on the NMs pathways, concerning the potential environmental impacts and relevant case studies, were included (Figure 3), which narrowed the number down to a total of 71 studies (Table 1). Studies in the last 19 years (2001–2020) were considered, to explore the trends of the LCA approach to NMs. The highest number of LCA studies was found in the year 2020.

### 2.2. Research Subject and Geographical Distribution of LCA Studies

**Figure 4:**
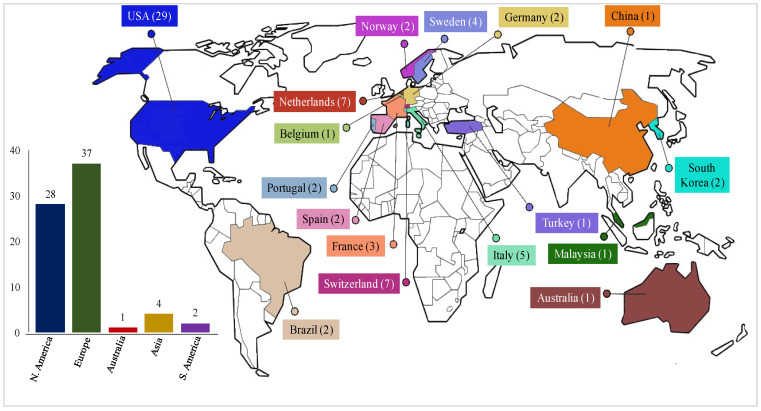
Total of published papers for each continent and country.

**Figure 5:**
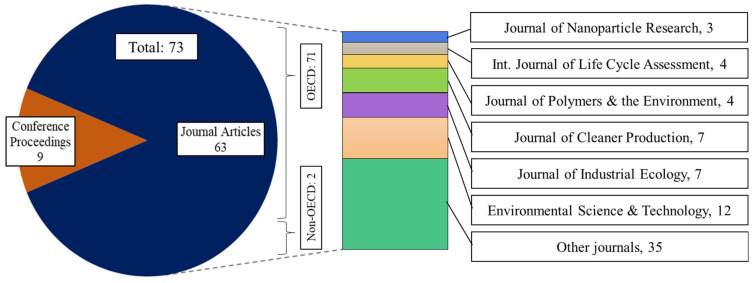
Distribution of the publications based on journals.

Figure 4 shows the total of published papers, based on the continent- and country-specific locations of the first affiliations of the publications. It was found that most of the studies were conducted in Europe (36 studies) and North America (28 studies), which represents about 95% of the published papers. Europe published the most articles in the year 2020 (8 articles out of 11). Moreover, the earliest article published on the LCA of NMs was also from Europe in 2001, indicating early investigation of the environmental impacts of NMs in this region. Figure 5 shows the distribution of reviewed papers, based on journals and types; Organization for Economic Co-operation and Development (OECD) and non-OECD countries. Most of the papers included are from OECD countries, accounting for 69 out of 71 studies.

#### 2.2.1. Functional Unit (FU)

The main goal of most of the reviewed studies was to evaluate the environmental impacts of nanomaterial products, from the beginning of the raw materials acquisition until the manufacturing and processing phases. The goal and scope definition phase in the LCA study represents the aim of the study, the product studied, its system boundary, and its functional unit (FU). The goal and scope definition addresses the strategies used to meet the assumptions made regarding NMs pathways. The FU is the quantified performance of NMs used as a reference unit in the LCA study, where a fixed value is set, and the output results on the environmental impacts from the impact categories reflect on this selected FU, where the margins of error and explicitly specified data uncertainties should be incorporated [90]. Table 1 shows that more than half of the reviewed studies used a simplified FU, relating to the weight of the material (e.g., 1 kg of a polymer nanocomposite). However, to perform a comparative LCA (e.g., comparing NMs with conventional materials), the FU should not be solely based on weight as it is not functionally comparable between the two products [11,91,92]. In fact, the FU should reflect the significance in evaluating the function and performance of such systems, where all processes, as well as their inputs and outputs, are linearly scaled [6,90]. A proper and adequate functional unit that considers all the additional functionalities of NMs must be considered in future studies in this field, to provide more realistic and fair potential benefits of nanomaterials in advanced technologies. Inadequate definition of the FU leads to higher uncertainty in the study [6,93].

#### 2.2.2. System Boundaries

The system boundary defines all operations that contribute to the life cycle of NMs, processes, and activities [10]. Case studies on metal, carbon, and composite nanomaterial products usually consider a cradle-to-gate LCA, including raw materials extraction and transportation of raw materials for product manufacturing, modification, and production of NMs until the use phase. However, the disposal stage and the potential toxicity of the product associated with the emissions of NMs during its life cycle are often neglected. This can be exemplified by the fact that a total of 55 out of 71 reviewed studies considered the system boundaries of cradle-to-gate; only 17 studies evaluated the entire life cycle of nanomaterials from cradle-to-grave.

A cradle-to-cradle approach is not commonly studied due to its complexity, which requires a re-utilization of materials in a more high-level view, based on the circular economy concept. This approach implies that the end of an NMs’ use cycle should be the raw material of another new process. Unfortunately, NMs have complex properties and compositions, which may change their physical–chemical interactions throughout the life cycle, especially at the disposal stage, making them challenging to repurpose into something new, since the NMs properties can be unpredictable [94]. It would be beneficial if further research could be carried out on the circularity use of NMs. Meanwhile, only [77] performed gate-to-gate by including the potential environmental impacts at the raw material acquisition stage, focusing mainly on evaluating and implementing strategies to improve the environmental status during this stage, without considering the other stages.

To date, there are no international regulations on the disposal management of NMs. As a result, most authors presumed that NM products are handled similarly to conventional products at the end-of-life stage. This factor contributes to a significant level of uncertainty around potential releases and consequences of NMs disposal management. Some studies that included the recovery or recycling stage considerably lowered the overall environmental impacts of the examined NMs, making them more enticing than conventional materials [13,66]. However, [79] stated that the final use and the end-of-life stages should also be included in the LCA study, as well as extending the system boundaries from cradle-to-grave, considering the final disposal of the produced NMs consist of combustion of the bio-organic product, which may have contributed to the potential environmental impacts. The study showed that midpoint indicators only reveal impacts somewhere between the emissions and the endpoint of NMs’ life cycle, while end-of-life stages are defined at the level of the protection areas (i.e., the environment, human health, and natural resources). Hence, it is crucial to consider the cradle-to-grave approach in future LCA studies, including the recovery and recycling stages, so that the potential impacts of studied NMs can be evaluated holistically.

### 2.3. Life Cycle Inventory (LCI)

The LCI phase, or known as the data collection phase, is crucial to any LCA study. This phase is the most work-intensive and time-consuming phase in an LCA, considering it requires a detailed data input of all the processes included in the scoping of NMs. Collections of complete and reliable data, which includes clear explanations of applied assumptions, advantages and disadvantages, and transparency and credibility criteria, are limited. The unavailable data needs to be covered by estimations, secondary, and generic data, which may lead to a higher level of uncertainty and limit the scope of the study [95].

Various up-to-date databases are available and can be used together with LCA software; however, those data (e.g., production of electricity, coal, or packaging) are generic and can only be used for processes that are not product-specific. Inventory database such as Ecoinvent is the most widely used by the researchers in the previous studies. The inputs in the inventory include raw materials, energy (renewable and non-renewable), and water, while outputs are the products and co-products—emissions to air, water, and soil [90]. Other databases such as BEES and ILCD are also used, depending on the study’s scopes and objectives that provide multicriteria fate modelling (such as USEtox, ReCiPe, and TRACI models) for evaluating the environmental impacts, fate, and exposure of certain products in the environment. Due to the limited inventory data availability, as shown by a few case studies in this review, several evaluated papers presented in Table 1 contribute to the inventory of NMs [92].

The majority of reviewed studies have 76% coverage on the input data (i.e., materials, energy, and water consumption during the synthesis and use of NMs). NMs flows in process outputs, on the other hand, are rarely stated, with just 18% of the studies reporting adequate data coverage for NMs emissions to environmental compartments and 28% reporting information on emissions from foreground processes. As can be seen from the percentages, inadequate data coverage is coming from the output side, revealing the scarcity of knowledge in NMs emissions throughout their life cycle. In the stages of NMs synthesis and manufacture, the foreground inventory data used in most of the reviewed studies are primarily drawn from secondary sources (literature) or lab-scale data. LCI data should be established individually for each life cycle stage in which NMs may be released, taking into account the type of matrix (the origin of NMs and its composition) as well as the nature of any transformation processes (alteration of properties) that may occur when NMs are released into the environment.

### 2.4. Life Cycle Impact Assessment (LCIA)

LCIA stage aims to assess the environmental impacts and analyze the data to evaluate the contribution to each impact category based on the inventory analysis, within the framework of the goal and scope established in the study [6,91,96]. This stage involves classification, characterization, normalization, evaluation, and weighing the data depending on the impact categories used in the study [78,91,97,98].

Most of the reviewed studies assessed the environmental performance of NMs up till the impacts on the midpoint level, such as eutrophication, acidification, ozone depletion, photo-oxidant formation, and climate change; only 26 reviewed studies extended the assessment of environmental impacts until the endpoint level of LCIA. The most studied impact categories are global warming potential (56%), acidification (38%), and ecotoxicity (36%). These major impact categories were studied frequently due to their significant impacts on the environment, disrupting the food chains by bioaccumulation, and eventually harming human health and all living things. As for these reviewed studies, the average value of global warming potential is between 0.7–3.0 kg CO_2_ eq per functional unit, while acidification produces a wide range of emissions, ranging from 1.0 to 20.0 kg SO_2_ eq per functional unit. For the ecotoxicity, on the other hand, the range is between 3.0–8.0 CTUe/kg, where the FU is in kg.

Ref. [55] only reviewed one impact category, which is ecotoxicity. The reason for this is that their main goal was to compare the relative impacts of carbon nanotubes production and exposure, using a shared metric of aquatic ecotoxicity, combined with toxicological studies. Ref. [70] also mentioned that impacts such as global warming potential and acidification do not mainly arise from the foreground system of NMs from graphene. Instead, they arise from background systems, such as transport and production of heat and electricity.

Ref. [78] evaluated the impacts at the midpoint level and categorized the potential impacts into eight categories. The results showed that NMs (nano-silica asphalt mixtures) performed better in global warming, ozone depletion, eutrophication, photochemical oxidation, and ecotoxicity than conventional asphalt mixtures. Other examples of reviewed studies that did not perform until endpoint level are [79,87], which evaluated 10–12 midpoint categories for the use of nanostructured materials in building blocks and nano-enhanced, carbon fiber-reinforced polymer prototypes, respectively. By using NMs rather than conventional materials, environmental impacts can be reduced, especially for climate change, photochemical ozone depletion, particulate matter (human health and ecosystem), and acidification.

**Figure 6:**
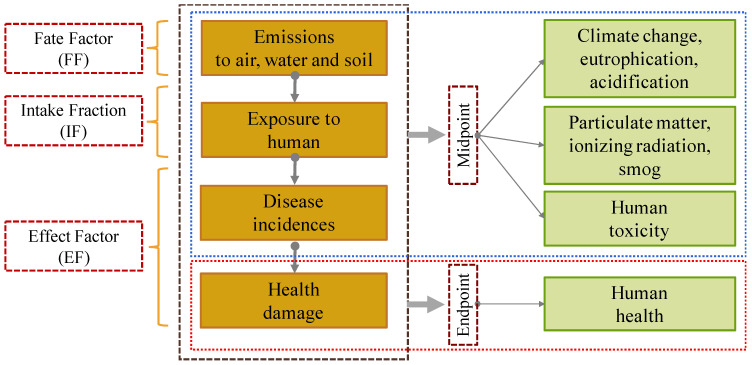
Toxicity flow of NMs.

Assessing the impacts up until the end-of-life stage, such as the disposal of NMs, was performed by a few studies using Eco-indicator 99 and ReCiPe [19]. A study was carried out by [12] on facade coating systems containing manufactured NMs, which included the endpoints level, where the disposal of the nano-titanium dioxide coatings was taken into account. It is proven that the studied manufactured NMs in coatings leads to an improved environmental performance, where the effects from long-term emissions in the final landfilling facilities have received almost no attention, but the dumping of unused paints containing NMs has to be reduced to the lowest level possible. LCIA at the end-of-life level is necessary for the LCA approach, but data availability is limited, hence increasing the study’s uncertainty. The human toxicity flow of NMs is shown in Figure 6.

#### 2.4.1. Fate Factor (FF)

Predicting the fate and behavior of NMs in the environment requires a specific understanding of the potential sources, distribution of NMs once it was released to the environment, transformation of NMs in the environment, and the persistence or adaptability of NMs in the environment [14]. There are still large uncertainties in each stage of modelling NMs transport by using existing models that cannot be quantified and are inconclusive [90].

Exploring the relative influences of the processes regarding NMs by their fate and behavior models can add considerable value to scientific efforts. However, the complete lifecycle, including the occurrence of releases of NMs and its fate in the context of ecological/environmental relevance, is one of the most critical issues missing in LCA studies published to date, relating to NMs and their applications [11,21,64,92,93,99]. Ref. [12] used the probabilistic material flow analysis (MFA) model as a fate model to support the complete life cycle. The probabilistic MFA model procedures were reported in [100]. In the study by [12], only a few articles assessed the pathways for the fate factor that includes the complete life cycle of the nanomaterials. A total of 26 articles analyzed the impacts until the endpoint; however, only a few included the fate factor analysis due to the complexity of the compounds, compositions, and chemicals contained in NMs. The large uncertainties for fate factor analysis remain untapped but exploring it would be significant in the scientific world. For example, [72] considered the fate factor and transformation of CeO_2_ nanoparticles during wastewater treatment and the role of hetero-aggregation in redox transformation; while [101] used a combined USEtox-SB4N approach to calculate the fate factor for unitary emissions of nano-TiO_2_ to air, freshwater, soil, and sediment.

#### 2.4.2. Exposure Factor (XF)

Ref. [55] was the first paper in the literature to consider XF using USEtox, which evaluated the NMs in the water column with assumed concentrations of suspended solids, dissolved organic carbon, and biota. Only five studies (7%) included the XF, which mainly focused on water bodies and bioaccumulation partitioning NMs out of the water column [13,55,69,75,76]. All studies stated that the value of XF is primarily based on assumptions and being considered as the worst-case scenarios, with the possible highest exposure taken into account. Thus, XF varies dramatically, depending on the input parameters of fate and transport of studied NMs.

Data on quantitative assessment of potential exposure of NMs are challenging to obtain and are currently scarce, where knowledge on its exposure mechanisms is limited. Data on the potential magnitude duration of NMs and the frequency of exposure are essential in determining environmental exposure assessment; however, the analytical measurements on concentrations of NMs in the environment have not yet been discovered, making it challenging to assess the accurate environmental exposure of NMs. In addition, distinguishing between manufactured NMs and naturally occurring NMs has been difficult, which is why this area remains a gap in this field. Although there are some existing models (e.g., MFA) that are often used to estimate the predicted environmental concentrations of NMs in geographical regions, those models pose high uncertainties in inputs and outputs, and lack a representative approach to validate the outcome [93,102,103].

Even in small amounts concerning NMs, evaluation of all critical aspects may potentially render adverse environmental effects. Moreover, certain NMs may undergo alternative disposal routes resulting in different exposure routes [102]. As mentioned in Section 2.2, most of the reviewed papers only studied the system boundaries in the cradle-to-gate stage; hence, the waste management systems of NMs are often neglected and remain as a gap. [102] demonstrated the estimation of nano-waste, which can be used for certain nano-products in which a variety of main aspects may be determined, and it was suggested that this could be carried out with the help of existing data. However, these estimations are associated with a considerable number of uncertainties, depending on the data quality. This information can provide a foundation for future research on the exposure of NMs in the environment.

#### 2.4.3. Effect Factor (EF)

The effect factor is derived from a list of published ecotoxicity studies. Most reviewed studies covered energy use, climate change, ecosystem quality, resources consumed, and human toxicity. The influence of NMs on the ecosystem, as determined by the EF, is based on toxicological data. For example, in freshwater ecotoxicity, the EF must be estimated using aquatic organisms’ chronic effective concentrations; while for human toxicity, the EF is measured using lethal or effective dosages reported for animals [21]. A total of 52 articles analyzed the impact categories, such as land use, eutrophication, acidification, environmental ecotoxicity (terrestrial, marine, and freshwater), greenhouse gases emissions, ozone depletion, and human toxicity [13,18,72,73,74,75,76,77,78,79]. Meanwhile, six articles assessed the effect factors using the TRACI method, four papers used Eco-indicator 99, and the rest used other methods in the LCA software [47,51,53,55,63,68,76].

Though many methodologies covered varieties of ecotoxicity and human health, the accuracy of the analysis still needs further improvement. In this case, the size of NMs makes it challenging to perform toxicity tests. The factors influencing the toxicity level of NMs include the size, thickness, surface layers, and surface functionalization [10,70,104]. Therefore, further direct chronic toxicity analysis on NMs is needed to improve the robustness and accuracy of effect factors.

### 2.5. Interpretation

The choice of an assessment tool to interpret the result plays a significant role in LCA studies because different frameworks provide different types of data and information [6,105,106]. Dubious results could be obtained if the selection of frameworks is inconclusive or interpreted out of the main contexts. Multicriteria decision analysis and decision theories such as comparative, sensitivity, and perturbation analyses can help interpret the results accordingly [6]. Interpretations within the cradle-to-gate and cradle-to-grave stages would be more definite and inconclusive with the combination of those decision–theory techniques, which are lacking in this area [107].

Most of the articles stated the possible improvements that could be made to reduce the environmental impacts of NMs. About 60% of the reviewed studies concluded that the environmental impacts could be reduced by optimizing the extraction stage, because the synthesis of NMs depends mainly on electricity production during the extraction stage. However, the use of lower temperatures may lead to a lower amount of NMs produced simultaneously. According to [68] the high impacts of producing carbon nanotubes are in the lithography stages, which include the lithography processes for trenches, contact leads (Pb), and metal deposition. The relative environmental impacts and human toxicity are consistent in both the midpoint and endpoint. However, the most significant environmental and human health impacts are still the energy consumed (in the form of electricity), accounting for 87% of the ozone depletion in the midpoint and over 50% to ozone depletion, particulate matter formation, human toxicity, and ionizing radiation in endpoint categories.

### 2.6. Advantages of Life Cycle Assessment Study

As mentioned, LCA allows better understanding of the potential environmental problems and ensure the environmental sustainability of NMs by assessing the environmental impacts of a product throughout its entire life cycle. Hence, adapting a comprehensive tool such as LCA will benefit any scientific studies to improve the environmental performance of a system. Correspondingly, LCA-based environmental evaluation is an integrated approach that can demonstrate whether NMs is a safe technology or vice versa. Furthermore, environmental effects can be quantified, such as energy consumption and air emissions, and by acknowledging the inefficiencies and drawbacks of a product (e.g., nanomaterials), scientists, product designers, service providers, and individuals would be enabled to make long-term decisions and improvements that take environmental aspects into account. LCA studies can also assist in analyzing significant shifts in environmental impacts throughout life cycle stages and its correlation to environmental releases. In terms of betterment in the NMs field, LCA can be beneficial to compare and study the human and ecological impacts between two or more rival products/processes, such as a study done by [86]. A conventional and proposed process in the production of nano-calcium carbonate (nCaCO_3_) was compared and found that by using the new proposed process design, the CO_2_ emission was reduced while remaining economically feasible.

## 3. Limitations and Uncertainties in LCA Study

### 3.1. Limitation of Current LCA Studies on Nanomaterials

Given that NM impacts may occur at any point of the life cycle, the end-of-life system boundary plays a big part in tackling the complete potential impacts, by virtue of NMs released throughout the usage and end-of-life stages are implausible to be in pristine form, unlike in the manufacturing and production stages. The limitations are that most authors assumed that NM products are handled similarly to conventional products at the end-of-life stages, due to limited knowledge on the flows of end-of-life NMs and the potential of emissions from various waste management processes (i.e., reuse and recycling). The same goes for LCI; the scarcity of data limits the accuracy of the environmental assessment by researchers. Some manufacturers do not disclose the materials and energy inputs for the production of NMs (commercial sensitivity), rendering data transparency low [6,13].

The lack of characterization factors in LCIA for nanomaterials is a primary concern. According to [93], to obtain relevant and representative characterization factors, a few critical aspects related to the risk assessment of NMs must first be addressed. These aspects include the following: (i) the fate of NMs (stressor) to the environment, (ii) the exposure of environmental receptors to the stressor, and (iii) the estimation of toxicity effect of the stressor on the environment. Considering these fundamental aspects of LCIA in future studies can improve understanding of NMs risks to the environment. Interpretations on the existing reported impacts on NMs being released to the environmental media only evaluate releases in their pristine form, which is not always a valid assumption. As mentioned in Section 2.4, NMs may undergo a transformative or ageing process that could change their properties to some extent in their life cycle, especially end-of-life stages. This interpretation and assumption lead to higher uncertainties in the assessment and limits the study’s accuracy. Only six studies included uncertainty analysis (Monte Carlo simulation), and most of them mentioned the difficulty of the analysis due to a vast gap between the current body of research and the number of toxicity studies.

### 3.2. Uncertainty of LCA for Nanomaterials

**Figure 7:**
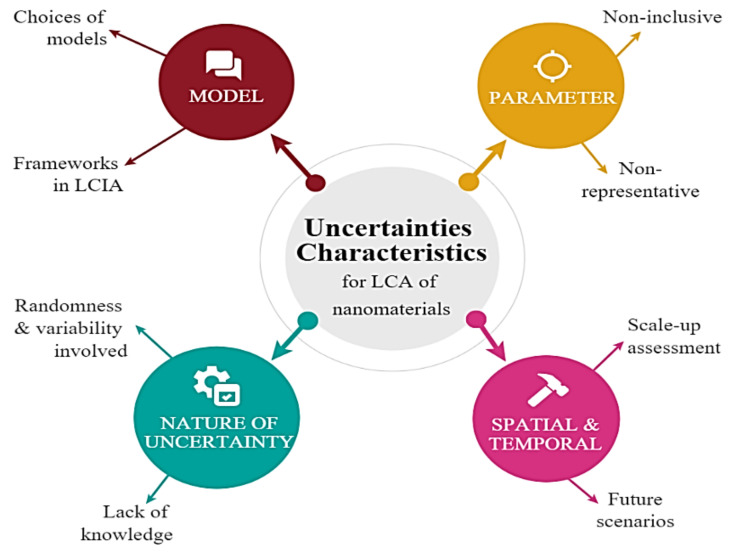
Uncertainties characteristics for LCA of nanomaterials.

There are a variety of limitations in LCA studies on NMs associated with their uncertainties. These uncertainties can be defined in various ways, but generally, uncertainty and variability are distinguished by model structure, parameter, spatial, temporal, and nature of uncertainty [93,108]. Identified uncertainties are characterized in Figure 7.

Uncertainties may come from the choices of models and frameworks while modelling, which leads to a lack of correlation between the mathematical models. In this case, data availability in released models should be improved by tackling the probabilistic distributions, while evaluating various distribution effects quantitatively and ranking the model’s output uncertainties. The results can only design simplified LCA models that focus on the environmental hotspots and main variables (environmental uncertainties). Parameters in LCA are vital; that is why some may have selected non-representative and non-inconclusive parameters. However, it can be improved by tackling the technology scale-up, processing, performance, and fate models in the environment and toxicity assessment, making it more representative in terms of emission values and potential impacts [108,109,110].

Uncertainty from the temporal/spatial representative may also exist, which involves scale-up assessment and future scenarios such as NMs releases and flows in the environment. Probabilistic MFA can reduce uncertainty in input values, but Bayesian networks can also be used depending on the data and input quality, which helps define the parameter and interrelationships through probability tables. Both are useful and flexible but have their drawbacks. For example, uncertainty uncertainties can cause a lack of knowledge or when randomness/variability is involved. Therefore, more research and efforts are required to tackle these problems for a more complete and comprehensive data collection and a higher model complexity [111,112].

Principally, the LCA framework is fully applicable to NMs technologies; however, despite this advantage, some critical uncertainties should not be overlooked when assessing LCA to support decision making around NMs. The first one is the lack of inventory data. Numerous LCA applied to NMs have been published to date, along with some papers that generally agree that many types of life cycle inventory are still unavailable. Manufacturers often do not adequately disclose the materials and energy inputs for commercial NMs due to commercial confidentiality. The same goes for acquiring data for the NMs emissions, for which, in most cases, the data are not measured by manufacturers or government entities during the production, use, and disposal stages [6,13].

Other than that, uncertainties in the inconsistency of laboratory data should not be overlooked either. Some researchers used different methods and approaches for different NMs, leading to changes in unit process data. Though the uncertainties of the laboratory data measurements could be estimated via Monte Carlo simulation, the inconsistency of the lab-scale methods and approaches suggest a certain quality of results and more detailed results that do not exist yet in such uncertainty analyses; therefore, uncertainty analysis would not provide any additional and detailed information at this stage. The development of characterization factors (i.e., fate, exposure, and effect factors) in NMs is still highly inadequate. This information is essential for released NMs for the life cycle impact assessment stage to make the impact assessment less inconclusive. [113] focused the study on the specific issue: the missing characterization factors for adequate LCIA analysis for release of NMs.

## 4. Recommendations and Future Prospect of LCA for Nanomaterials

There is a rapid growth in research and application of NMs, especially in Asia, due to their multi-functionality and urgent need for environmental, human health, and safety. As a result, many scientific studies on the LCA of NMs have been published, which regulatory and industrial stakeholders can refer to when making decisions regarding their products development and assessment methods. However, LCA studies on NMs are currently affected by the knowledge gap respecting the release and exposure of NMs into the environment. Based on the findings, Table 2 provides recommendations to LCA practitioners working in NMs or nanotechnology on improving the gaps in consistency, transparency, and completeness.

As mentioned throughout the review, NMs have become an emerging technology worldwide, especially in Southeast Asian countries, notably in textiles, healthcare, and biomedical fields [3,114]. Along with rising needs and usage that may lead to higher toxicity, toxicity assessments for NMs are crucial in evaluating the exposure pathway and analyzing how substantial the impacts of NMs are on the environment and human health. On top of that, Asian countries that used NMs technologies in most of their products, especially South Korea and Japan, outperform countries in other regions in healthcare performance; thus, the potential of nanotechnology in Southeast Asian countries is vast [115,116]. Thus, by collaborating with these manufacturers and developers, researchers would be more efficient in analyzing the rather time-consuming materials flow identification in the production, release, and exposure of NMs.

## 5. Conclusions

Most of the published studies strive to address some of the challenges and limitations. It was found that most of the main issues identified are related to the inadequate definition of functional unit, insufficient LCI datasets that are high quality and relevant, and lack of characterization factors for NMs emissions, specifically in toxicity assessment. Due to these limitations, most studies concerning NMs in the life cycle are inconclusive, except those with specific product systems studied. These shortcomings and gaps remain unresolved unless stated otherwise in future studies. Some recommendations have been put forth regarding those issues; one of which is increasing the efforts to assess the environmental impacts and potential risks of NMs within their whole life cycle, including the end-of-life stages. Further research is also needed to fill the gaps in the relevancy of high-quality data inputs and outputs and develop more complex practical and analytical methodologies for fate, transport, toxicity, sensitivity, and impact studies.

We underlined the significance of thorough uncertainty analyses and assessments of LCA in general, particularly in the studies of NMs. Adequate use of transparent and complete characterization model during the interpretation phase of LCA requires extra efforts from the LCA practitioners and researchers, and toxicity studies should be explored more widely for a comprehensive and reliable LCA study. Since healthcare products that use NMs are in future growth, especially in Asian countries such as South Korea and Japan, toxicity assessment for NMs is crucial to evaluate the exposure pathways and analyze how substantial the impacts of NMs are on the environment and human health. Among the various initiatives researchers take in this field, the LCA approach to NMs is essential and offers engaging results to improve the environmental profile and hotspot.

## Figures and Tables

**Table 1:** Peer-reviewed LCA studies on NMs.

No.	Reference	Type of Nanomaterials	Method/Software	Impact Categories	System Boundaries	Functional Unit	Impact Assessment	
							Mid-Point	Endpoint
1.	[26]	Nanocrystalline	Ecoindicator 95, Environmental Priority Strategies (EPS), Eco Sweden, Eco Netherlands and Environmental Design of Industrial Products (EDIP)/SimaPro	Greenhouse gases (GHG) emissions, air emissions, electricity generation	Cradle-to-grave	1 kWh electricity	O	O
2.	[27]	Nanoclay polymer composites	Economic Input-Output Life Cycle Assessment (EIO-LCA) through Economic Input-Output (EIO) model/GaBi 4	Projected fuel savings, Carbon Dioxide (CO_2_) reduction, economic inputs and outputs, GHG emissions, toxic releases	Cradle-to-grave	16.9 million light-duty vehicles, 210 million vehicles on the road	O	O
3.	[28]	Nanoscale platinum-group metal particles	EIO-LCA through EIO model/GaBi 4	Economic inputs and outputs, economic purchases, emissions of conventional pollutants and greenhouse gases, RCRA hazardous waste, toxic releases	Cradle-to-gate	Projected motor vehicles in the US between 2005 and 2030	O	O
4.	[29]	Various oxide nanoparticles	Not stated - Ecoinvent	Energy consumption, CO_2_ emissions	Cradle-to-gate	1 kg	O	X
5.	[30]	Single-walled carbon nanotubes	EPS 2000/SimaPro	Human health, production capacity, abiotic resources, biodiversity	Cradle-to-gate	1 g	O	X
6.	[31]	Nanoclay polypropylene layered silicate nanocomposite packaging film	Not stated/SimaPro Derived from the latter: energy and material data from the pilot plant	Non-renewable energy use (NREU), GHG emissions	Cradle-to-grave	1000 bags	O	X
7.	[31]	Nanoclay polypropylene layered silicate nanocomposite agricultural film	Not stated/SimaPro Derived from the latter energy and material data from the pilot plant	GHG emissions	Cradle-to-grave	Coverage of 650 m^3^	O	X
8.	[31]	Nanoclay polypropylene layered silicate nanocomposite automotive panels	Not stated/SimaPro Derived from the latter energy and material data from the pilot plant	NREU, GHG emissions, abiotic depletion, ozone layer depletion, photochemical oxidant formation, acidification, eutrophication	Cradle-to-grave	Internal panel of low-weight family car over 150,000 km operation	O	X
9.	[32]	Nanoscaled organophilic montmorillonite in PHB fillers	Not stated PlasticsEurope LCA database used	GHG emissions and NREU	Cradle-to-grave	17-inch CRT monitor	O	O
10.	[33]	Single-walled carbon nanotubes	Not stated	Human exposure	Cradle-to-grave	1 kg	O	X
11.	[34]	Nanoscale semiconductor fabrication and manufacturing	EIO-LCA through EIO model/SimaPro Primary data used	Economic inputs and outputs, GHG emissions (uncertainty included)	Cradle-to-gate	1 wafer with 300 mm diameter	O	X
12.	[35]	Nanoclay biopolymer composites	Not stated Ecobilan’s Data for Environmental Analysis and Management (DEAM)™ LCA database used	Energy demand and GHG emissions, non-renewable energy savings	Cradle-to-gate	1 kg	O	X
13.	[36]	Carbon nanofibers	Not stated/SimaPro Industrial data of the United States (US) economy for the 20th century and US LCI database used	Energy analysis, GHG emissions, human toxicity potential (sensitivity analysis included)	Cradle-to-gate	1 kg	O	O
14.	[37]	Fullerenes and single-walled carbon nanotubes	Not stated PlasticsEurope, LCA database used	Energy consumption, carbon yield	Cradle-to-gate	1 kg	O	O
15.	[38]	Single-walled carbon nanotubes	Not stated/SimaPro HiPco model inventory used	Climate change, airborne inorganics, acidification	Cradle-to-gate	1 g	O	O
16.	[39]	Carbon nanofibers—Polymer nanocomposites	Not stated PlasticsEurope, LCA databases used	GHG emissions and impact (toxicity impact included)	Cradle-to-gate	Midsize car over 150,000 miles of operation	O	O
17.	[40]	Nanotitanium dioxide photocatalyst coatings for concrete pavement	EIO-LCA/SimaPro	Economic inputs and outputs, acidification, eutrophication, criteria air pollutants, smog formation	Cradle-to-gate	1 km lane of pavement	O	X
18.	[41]	Vapor-grown carbon nanofibers, polymer nanocomposites	Collected from values reported in literature and LCA software/SimaPro	Energy consumption (sensitivity and uncertainty analysis included)	Cradle-to-grave	1 kWh electricity generated	O	X
19.	[42]	Yttria-stabilized zirconia, nanostructured coating	EDIP 2003/SimaPro Ecoinvent database used	Ozone depletion potentials, GHG emissions, eutrophication, human toxicity, ecotoxicity, hazardous waste, slags/ashes, bulk waste, radioactive waste, resources	Cradle-to-gate	1 micrometer thick area of 1 m^2^ surface	O	O
20.	[43]	Titanium dioxide nanoparticles	Eco-indicator 99/SimaPro	Carcinogen, climate change, GHG emissions, radiation, ozone layer, acidification, land use, airborne organics and inorganics, (uncertainty analysis included)	Cradle-to-gate	1 kg	O	O
21.	[44]	Single-walled carbon nanotube	EIO-LCA through EIO model/SimaPro	Economic inputs and outputs	Cradle-to-gate	1 kg	O	X
22.	[45]	Nanoelectronics, multi-walled carbon nanotube	Chain Management by Life cycle assessment (CML)/Umberto Ecoinvent database used	Energy consumption	Cradle-to-gate	1 kg	O	X
23.	[46]	Quantum dot photovoltaics	Impact 2002+/SimaPro Ecoinvent database used	Energy consumptions, lower GHG emissions, SO_x_, NO_x_ emissions	Cradle-to-gate	1 kg	O	O
24.	[47]	Silver nanoparticles	Tool for Reduction and Assessment of Chemicals and Other Environmental Impacts (TRACI) 2.0 v-3.01 and EIO-LCA model/SimaPro	Economic inputs and outputs, GHG emissions, acidification, carcinogens, euthrophication, ozone depletion, ecotoxicity	Cradle-to-gate	1 mg	O	O
25.	[48]	Nanosilver t-shirts	USES-LCA/SimaPro Ecoinvent database used	GHG emissions, freshwater toxicity, waterborne emissions (sensitivity and uncertainty analysis included)	Cradle-to-gate	1 kg	O	X
26.	[49]	Nano-crystalline materials in thin-film silicon solar cells	Not stated/Simapro Ecoinvent database used	Climate change, ozone depletion, GHG emissions, acidification, ecotoxicity, human toxicity (toxicity impact included)	Cradle-to-gate	1 m^2^ of module area and 1 kWh	O	X
27.	[50]	Single-walled carbon nanotubes	EIO-LCA model /SimaPro HiPco data used	Economic inputs and outputs, energy consumption	Cradle-to-gate	1 kWh	O	X
28.	[51]	Starch nanocrystals	TRACI 2 and Ecoindicator 99/SimaPro	GHG emissions, acidification, climate change, radiation, ozone layer, ecotoxicity, land use, respiratory organics and inorganics	Cradle-to-gate	1 kg and 10,000 m^2^ of packaging material	O	O
29.	[52]	Black carbon and activated carbon with single-walled and multi-walled carbon nanotubes	Primary data in laboratory-scale study/not stated	Energy consumption	Cradle-to-gate	1 MJ/kg	O	X
30.	[53]	Nanoparticles coated recovered fiber paper	ReCiPe, Building for Environmental and Economic Sustainability (BEES), Life Cycle Cost (LCC) and TRACI/SimaPro Ecoinvent and BEES databases used	Energy consumption, NREU, renewable energy use (REU), GHG emissions	Cradle-to-grave	1 tonne	O	O
31.	[54]	Cellulose nanowhiskers	ReCiPe/SimaPro Ecoinvent database used	Climate change, water depletion, eutrophication, human toxicity	Cradle-to-gate	1 g of cellulose nanowhiskers	O	X
32.	[55]	Carbon nanotubes	USEtox model/SimaPro	Ecotoxicity (uncertainty analysis included)	Cradle-to-gate	1 kg of carbon nanotubes	O	X
33.	[56]	Molybdenum sulfide (MoS_2_) nanoparticles	Not stated/SimaPro Ecoinvent database used	Energy consumption, GHG emissions	Cradle-to-gate	1 g of MoS_2_ nanoparticles	O	O
34.	[57]	Organic photovoltaics from nanomaterials	Not stated/SimaPro Ecoinvent database used	GHG emissions, energy consumption, acidification, ozone depletion potential, human toxicity, ecotoxicity	Cradle-to-grave	1 kg	O	O
35.	[58]	Carbon nanotubes	TRACI and primary data/SimaPro Ecoinvent database used	GHG emissions, acidification, GHG emissions, eutrophication, ozone depletion, smog formation, ecotoxicity, human health, respiratory effects	Cradle-to-gate	1 unit of Si wafer with a surface area of 45 cm^2^ and 4 g mass	O	X
36.	[59]	Nano-sized titanium dioxide coatings	BEES 4.0 model/BEES software	Acidification, eutrophication, air pollutants and smog formation potential, GHG emissions, fossil fuel depletion, water intake, human health, ecological toxicity	Cradle-to-gate	1 m^2^ of titanium dioxide-coated glass	O	X
37.	[60]	Nano-coated wooden claddings	ReCiPe, Europe Ecolabel (EU-Ecolabel) /SimaPro 7.3 Ecoinvent database used	Air emissions, water emissions	Cradle-to-grave	0.01 m^2^ of coated exterior wooden cladding	O	O
38.	[61]	Hollow silica nanospheres, nano insulation materials	Primary data in laboratory-scale study/not stated	Energy consumption	Cradle-to-gate	1 g of hollow silica nanospheres	O	X
39.	[62]	Nanocellulose	Eco-Indicator 99/SimaPro	Energy consumption, carcinogens, human health respiratory organics and inorganics climate change, GHG emissions, radiation, ozone layer, ecotoxicity, acidification, eutrophication, land use resources	Cradle-to-gate	10 g equivalent dry mass of the end product nanocellulose	O	X
40.	[63]	Silver nanoparticles bandages	TRACI/SimaPro Ecoinvent database used	Ozone depletion, GHG emissions, smog formation, respiratory effects, water and soil quality impacts, acidification, eutrophication, human health, ecotoxicity	Cradle-to-grave	1 g	O	O
41.	[64]	Carbon nanotubes field emission displays (CNT-FEDs)	TRACI, USEtox/SimaPro Ecoinvent and National Renewable Energy Laboratory (NREL) US LCI database used	GHG emissions, acidification, human health, carcinogens and noncarcinogens. respiratory effects, eutrophication, ozone depletion, ecotoxicity, fossil fuel depletion, ecotoxicity	Cradle-to-grave	10,000 viewing hours	O	O
42.	[65]	Cellulose nanocrystals/cellulose nanofibrils from wood pulp	TRACI and primary data of pilot-scale production/SimaPro Ecoinvent database used	Energy consumption, GHG emissions, ozone depletion, acidification, eutrophication, human health, ecotoxicity, fossil fuel depletion	Cradle-to-gate	1 kg of cellulose nanocrystals	O	X
43.	[66]	Gold nanoparticles (AuNP)	Not stated/SimaPro Ecoinvent database used	Energy consumption, climate change, metal depletion, agricultural land occupation, freshwater ecotoxicity	Cradle-to-gate	1 mg of AuNP	O	O
44.	[67]	Graphite nanoplatelets (GnP)	ReCiPe, USEtox, EDIP,CML/SimaPro Ecoinvent database used	Energy consumption	Cradle-to-grave	1 kg of epoxy composite loaded with 0.058 kg of GnP	O	O
45.	[68]	Carbon nanotube-enabled chemical gas sensor	ReCiPe and TRACI 2/SimaPro Ecoinvent database used	GHG emissions, acidification, eutrophication, ozone depletion, smog formation, human health impacts from carcinogenic, noncarcinogenic, respiratory disease, ecotoxicity	Cradle-to-gate	1 g per chip	O	O
46.	[69]	Single-walled carbon nanotubes, multi-walled carbon nanotubes	USEtox model/Microsoft Excel	Human toxicity, freshwater ecotoxicity	Cradle-to-gate	1 nm	O	X
47.	[70]	Nanomaterials from graphene	USEtox model/not stated	Energy use, water use, human toxicity, ecotoxicity, (sensitivity analysis included)	Cradle-to-gate	1 kg of graphene in solution	O	X
48.	[71]	Nano insulation materials consisting of hollow silica nanospheres	Not stated Primary data in laboratory-scale study used	Energy consumption	Cradle-to-gate	1 g	O	X
49.	[72]	Cerium Dioxide (CeO_2_) nanoparticles	Monte Carlo/mathematical modeling software	Toxicity and uncertainty analysis	Cradle-to-gate	1 tonne	X	O
50.	[73]	Cellulose nanofibrils from wood pulp	ReCiPe /not stated Ecoinvent database used	Energy use, climate change, acidification, water use (sensitivity analysis included)	Cradle-to-gate	1 kg	O	X
51.	[12]	Titanium dioxide, silver and silica nanoparticles in facade coatings/paints	ReCiPe and USEtox/Open LCA tool Ecoinvent database used	GHG emissions, freshwater eutrophication, fossil fuel depletion, acidification, ecotoxicity, human toxicity, human health, resource availability	Cradle-to-gate	1 square meter of (indoor or outdoor) wall during 80 years	O	O
52.	[74]	Tungsten disulphide nanoparticles	ReCiPe, CML and primary data collection from an industrial process/SimaPro Ecoinvent database used	Energy resources, GHG emission, acidification, euthrophication, human toxicity	Cradle-to-gate	1 g	O	X
53.	[75]	Graphene oxide nanomaterial	USEtox and ReCiPe/SimaPro	Freshwater ecotoxicity (sensitivity analysis included)	Cradle-to-gate	1 kg	O	X
54.	[76]	Silver nanoparticles	TRACI and USEtox model/SimaPro	Ozone depletion, GHG emissions, photochemical smog formation, acidification, eutrophication, carcinogens, air pollutants, ecotoxicity, fossil fuel depletion	Cradle-to-grave	1 kg	O	O
55.	[77]	Printed electronic temperature sensor composed of specialized carbon nanotube	IMPACT 2002+ model/Simapro Ecoinvent database used	Carcinogens, respiratory organics and inorganics, ionizing radiation, ozone layer depletion, ecotoxicity, GHG emissions, land occupation, NRE, mineral extraction	Gate-to-gate	2400 sensors/day	-	O
56.	[18]	Nano-scale zero valent iron	IMPACT 2002+/SimaPro Ecoinvent database used	Climate change, ecosystem quality, human health, resources	Cradle-to-gate	1 g	O	X
57.	[13]	Nano-titanium dioxide	USEtox/SimpleBox4Nano	GHG emissions, freshwater eutrophication, fossil fuel depletion, acidification, ecotoxicity, human toxicity, human health	Cradle-to-gate	1 nm	O	X
58.	[78]	Nano-silica-modified asphalt mixtures	TRACI/Open LCA tool—Ecoinvent database used	Ecotoxicity, carcinogens, GHG emissions, ozone depletion, acidification, eutrophication, respiratory effects	Cradle-to-gate	1000 kg production of nano-silica-modified asphalt mixtures	O	X
59.	[79]	Cellulose nano-sponges	International Reference Life Cycle Data System (ILCD) 2011 Midpoint+/SimaPro Ecoinvent database used	Climate change, ozone depletion, human toxicity, GHG emissions, particulate matter, ionizing radiation, photochemical ozone formation, acidification, eutrophication, freshwater ecotoxicity, water resource depletion, renewable resource depletion	Cradle-to-gate	1 kg of cellulose nanosponge	O	X
60.	[10]	Nano-wire based solar cells	Primary data in laboratory-scale/SimaPro Ecoinvent database used	Land use, eutrophication, acidification, GHG emissions, photochemical oxidation, climate change, ecotoxicity, ozone depletion, human toxicity	Cradle-to-gate	1 kWh of electricity production	O	X
61.	[80]	Nano-scale zero-valent iron	IMPACT 2002+/Simapro Ecoinvent database used	Energy consumption, human health, atmospheric emissions	Cradle-to-gate	1 kg	O	X
62.	[81]	Binary oxides nanoparticles	TRACI 2.1/SimaPro Ecoinvent and US Life Cycle Inventory used	Ozone depletion, GHG emissions, smog, acidification, eutrophication, carcinogenic and noncarcinogenic, respiratory effects, ecotoxicity, fossil fuel depletion	Cradle-to-gate	1 kg	O	X
63.	[82]	Photo-Fenton catalysts with combinations of magnetite nanoparticles semiconductor	IMPACT and ReCiPe/SimaPro Ecoinvent database used	Climate change, ozone depletion, acidification, eutrophication, toxicity, fossil depletion	Cradle-to-gate	1 kg	O	X
64.	[83]	Fly ash hydrated lime blended concrete nanosilica	Not stated/SimaPro Ecoinvent database used	GHG emissions, acidification, photochemical oxidant formation impact	Cradle-to-gate	kg/m^3^	O	X
65.	[84]	Nano-powder in glass bottle wastes	Not stated Primary data in laboratory-scale study used	CO_2_ emission, energy consumption, fuel consumption	Cradle-to-gate	1 m^3^	O	X
66.	[85]	Nano-hydroxyapatite	IMPACT/SimaPro	GHG emissions, non-renewable energy, respiratory inorganics, human health, climate change, resources, ecosystem quality	Cradle-to-gate	10 g	O	O
67.	[86]	Nano calcium carbonate	Not stated/SimaPro Ecoinvent database used	GHG emissions, CO_2_ emissions (sensitivity analysis included)	Cradle-to-gate	1 g	O	X
68.	[87]	Nano-enhanced carbon fiber-reinforced polymer	ILCD Midpoint +/SimaPro Ecoinvent database used	Human toxicity, respiratory effects, ionizing radiation, photochemical oxidation, climate change, ozone depletion, GHG emissions, human health, ecotoxicity, acidification, eutrophication, land occupation, water consumption, NRE, mineral extraction, water turbined	Cradle-to-gate	1 product piece	O	X
69.	[88]	Silver nanomaterials	TRAP (Toxicity Relationship Analysis Program)/REST-MSC tool	Water and soil emissions	Cradle-to-gate	mg/kg	O	X
70.	[17]	Engineered nanomaterials	In vivo No-Observed-Adverse-Effect Level (NOAEL), Lowest-Observed-Adverse-Effect Level (LOAEL), EC_50_ or ED_50_ (Effective Dose/Dosage) methods/not stated	Human health, human toxicity	Cradle-to-gate	1 kg	O	X
71.	[89]	Nano-grid	ReCiPe/OpenLCA tool Ecoinvent database used	Ecotoxicity, human health, resources (sensitivity analysis included)	Cradle-to-grave	1 MWh	O	X

“O” indicates that the corresponding life cycle phase was assessed in the study (qualitative/quantitative). “X” indicates that the corresponding life cycle phase was not assessed in the study.

**Table 2:** Limitations and recommendations to LCA practitioners in the field of NMs.

No.	Limitations	Uncertainties	Possible Approaches/Recommendations
1.	Scarcity of knowledge: End-of-life stages. Potential of emissions from various waste management processes.	Uncertainties in outputs, final emissions, and interpretation stage.	Incorporate complete information on NMs properties into existing tools to enhance fate, behavior, and the impacts of NMs. Further research is needed to improve the understanding of physical and chemical changes in properties for eventual releases.
2.	Inadequate data on LCI.	Uncertainties in process inputs, outputs, and final emissions.	Combining LCA-RA approach. All NMs life cycle emissions must be taken into account in a manner as complete and transparent as possible.
3.	Lack of characterization factors in LCIA.	Uncertainties in fate, exposure, and effect factors.	The use of a transparent and prudent characterization model is still highly recommended. Includes toxicity and sensitivity assessments to analyze the exposure pathways of NMs further.
4.	Invalid assumptions in the interpretation stage.	Uncertainties in results being irrelevant and unclear conclusions if interpreted out of context.	Incorporate complete literature data on NMs properties, full assessments, and analyses on the entire life cycle.
